# Walking the Tightrope: A Proposed Model of Chronic Pain and Stress

**DOI:** 10.3389/fnins.2020.00270

**Published:** 2020-03-26

**Authors:** Claire E. Lunde, Christine B. Sieberg

**Affiliations:** ^1^Department of Psychiatry, Boston Children’s Hospital, Boston, MA, United States; ^2^Biobehavioral Pediatric Pain Lab, Boston Children’s Hospital, Boston, MA, United States; ^3^Center for Pain and the Brain (P.A.I.N. Group), Department of Anesthesiology, Critical Care and Pain Medicine, Boston Children’s Hospital, Boston, MA, United States; ^4^Nuffield Department of Women’s and Reproductive Health, Medical Sciences Division, University of Oxford, Oxford, United Kingdom; ^5^Department of Psychiatry, Harvard Medical School, Boston, MA, United States

**Keywords:** chronic pain, chronic stress, allostatic load, physiology, behavior, reactivity

## Abstract

Pain and stress are both phenomena that challenge an individual’s homeostasis and have significant overlap in conceptual and physiological processes. Allostasis is the ability to adapt to pain and stress and maintain homeostasis; however, if either process becomes chronic, it may result in negative long-term outcomes. The negative effects of stress on health outcomes on physiology and behavior, including pain, have been well documented; however, the specific mechanisms of how stress and what quantity of stress contributes to the maintenance and exacerbation of pain have not been identified, and thus pharmacological interventions are lacking. The objective of this brief review is to: 1. identify the gaps in the literature on the impact of acute and chronic stress on chronic pain, 2. highlight future directions for stress and chronic pain research; and 3. introduce the Pain-Stress Model in the context of the current literature on stress and chronic pain. A better understanding of the connection between stress and chronic pain could provide greater insight into the neurobiology of these processes and contribute to individualized treatment for pain rehabilitation and drug development for these often comorbid conditions.

## Introduction

The objective of this brief review is to: 1. identify the gaps in the literature on the impact of acute and chronic stress on chronic pain, 2. highlight future directions for stress and chronic pain research; and 3. introduce the Pain-Stress Model in the context of the current literature on stress and chronic pain.

### Chronic Pain

Defined as pain lasting longer than 3 months after the resolution or in the absence of an injury (Treede et al., 2015), chronic pain is a significant humanitarian burden affecting 11–40% of adults (Dahlhamer et al., 2018) and 5–38% of youth (King et al., 2011). Chronic pain has been associated with constraints in mobility and daily activities, dependence on opioids, increased levels of anxiety and depression, poor perceived health, and a reduced quality of life (Gureje et al., 1998; Smith et al., 2001; Dahlhamer et al., 2018). Different types of pain (chronic, acute, nociceptive, neuropathic, etc.), result in different modulation of each other, which leads to different consequences (Crofford and Casey, 1999). This brief review will focus solely on chronic pain and its complex and dynamic relationship to different types of psychological stress.

### Stress

Costing over 300 billion dollars annually (Mucci et al., 2016), chronic stress is categorized as a “worldwide epidemic” by the World Health Organization, and has been associated with increased rates of mental illness and suicide (Rizvi et al., 2017). Although anxiety and stress are similar (Andolina et al., 2016; Ray et al., 2017), psychological stress is less clearly defined than anxiety disorders and as a result, there is not a recognized pharmacological treatment for stress. A review (Shekhar et al., 2005) summarized data supporting the hypotheses that stress induced plasticity within the amygdala may be a critical step in the pathophysiology of the development of chronic anxiety conditions. This evidence suggests that anxiety is a reaction to stress, however, both have been investigated as interchangeable mechanisms. Stress-related psychological conditions, such as anxiety, are frequently associated with pain (Felice et al., 2015; Luisi et al., 2015; O’Mahony et al., 2017), with multiple biological processes contributing to allostatic overload and influencing the pathophysiology of the central nervous system (CNS) (McEwen, 1998, 2000, 2005; Crimmins et al., 2003; Brotman et al., 2007; McEwen and Gianaros, 2011; Karatsoreos and McEwen, 2013). Chronic pain is one potential manifestation of this allostasis.

### Link Between Chronic Pain and Stress

Chronic pain is highly comorbid with chronic stress (Davis et al., 2017). Anxiety, depression, and pain catastrophizing (i.e., excessive worry about pain) have been associated with the presence of chronic pain and with a poor prognosis in people with a wide array of pain conditions (Baum, 1990; van der Windt et al., 2002; McWilliams et al., 2003; Boersma and Linton, 2006). Many studies examining risk factors for pain report an association between musculoskeletal pain and pain-related psychosocial stress, including fear, catastrophizing, and negative coping (Linton, 2000; George and Stryker, 2011; Lumley et al., 2011; Crombez et al., 2012; Lucchetti et al., 2012). Exaggerated, prolonged, or recurrent activation of a sensitized stress response to pain or non-pain-related stressors may initiate or exacerbate chronic pain and disability (Heim et al., 2000; Tak and Rosmalen, 2010; Hall et al., 2011). Two existing theoretical models linking pain and stress include: 1. stress overload leads to the onset and persistence of chronic pain. Pain is one *type* of stress that adds strain on an individual which results in allostatic overload in the body and brain from chronic impairment in the regulation of physiological systems that are historically involved in adaptation to environmental challenges (Vachon-Presseau et al., 2013) and 2. allostatic overload *triggers* or induces chronic pain. Focusing on the long-term consequences, unforeseeable stress induces pain and a cycle of “feeding-forward” inadequate adjustments to the situations, thus resulting in physiological responses and susceptibility to pain persistence (Borsook et al., 2012). Both models emphasize that stress and pain are interconnected and are two components in a cycle of maladaptive responses to challenging environmental situations.

The body and the brain have substantial capacity for adaptive plasticity; however all exposures to stress are not necessarily irreversible (Korte et al., 2005). Studies (Schneiderman et al., 2005) also indicate that there may be differences in susceptibility to environmental stressors on three levels: behavioral, physiological, and genetic. A third model which is not specific to pain and stress but applicable to elucidating their potential relationship concerns *biological reactivity*. Specifically, the relationship between early adverse life events and development of an individual’s *reactivity* to stress is curvilinear, with high reactivity phenotypes emerging in both high stress and protected early social surroundings (low stress) (Boyce and Ellis, 2005). Individuals exposed to very low adversity may develop high biological sensitivity to take maximum advantage of positive environmental influences. This model supports recent findings from our group (Sieberg et al., 2018). We examined the effects of lifelong mild stress on levels of neuropathic pain and the effects of chronic neuropathic injury on anxiety-like behavior in mice with results demonstrating a strong link between chronic neuropathic pain and chronic anxiety (Soria et al., 2015), with the driver of this comorbidity being neuropathic pain as opposed to ongoing stress (Sieberg et al., 2018). However, the timing, severity, and type of stress needed to induce this cruel cycle is unclear, and alternatively, if there is a healthy or protective dose of stress that could buffer or prevent the onset of chronic pain, or how much stress is protective is also unknown.

## Mouse Models of Chronic Pain and Stress

Heightened risk of pain and psychological stress are often co-morbid in clinical studies (Noel et al., 2016; Sachs-Ericsson et al., 2017; Lo Curto et al., 2019; Beal et al., 2020), however, animal studies yield contradictory results about the nature of their interaction. Specifically, it has yet to be established in neither clinical nor animals studies if psychological distress is a precursor (Rivat et al., 2010) to or consequence (Eccleston and Clinch, 2007) of living with chronic pain.

### Overview of Existing Literature

Liu et al. (2015) found that persistent inflammatory pain and social defeat stress-induced anxiety may not exacerbate one another in animal models of co-morbidity. Aizawa et al. (2018) showed that the role of G protein-coupled receptor 40/free fatty acid receptor 1 is a signal for the development of chronic pain and is induced by emotional dysfunction (Aizawa et al., 2018). These findings indicate that this specific impairment of regulation of this protein and fatty acid signaling the brain underlying stress conditions is directly related to the development of chronic pain. The same group of researchers also found that repeating the administration of naloxone exacerbated mechanical allodynia due to postoperative pain. This suggests that the underlying mechanism for pain exacerbation induced by inhibition of the G protein-coupled receptor 40/free fatty acid receptor 1 may be associated with naloxone-induced exacerbation of postoperative pain (Nakamoto et al., 2017). Using a chronic pain mouse model, Wang et al. (2017) investigated individual variance in two dimensions of pain behaviors: sensory and emotional. Results showed that mice displayed heterogeneous sensitivities in the chronic pain-induced anxiety- and depression-like behaviors of affective pain. Additionally, their molecular analyses revealed that the mice with higher vulnerabilities to developing emotional disorders, such as depression and anxiety, also revealed to have lower levels of protein in the amygdala, and more specifically, in the emotion-processing central nucleus (Wang et al., 2017). Their findings suggest that individual vulnerabilities to pain may be ingrained in the emotional aspect of chronic pain and remain consistent in aspects of negative emotions, in which adaptive changes in the role of the changed protein levels in central amygdala may have significant and long-term consequences.

A behavioral experiment conducted by our group (Sieberg et al., 2018) found that 1. the effects of long-term spared nerve injury (SNI) and life-long stress are not markedly different to SNI alone, suggesting that ongoing pain derived from nerve injury is the primary driver of the prominent anxiety-like phenotypes witnessed in the mice examined and 2. long-term SNI and chronic stress were almost equivalent in increasing plasma corticosterone levels, which suggests similar levels of signaling through this endogenous stress system for each condition. Glutamate is known to be the primary excitatory neurotransmitter modulating nociceptive networks, and Glt1 is critical in pain signaling termination (Greenwood-Van Meerveld and Johnson, 2017). Chronic emotional stress results in hyperalgesia that correlates with altered CNS glutamate processing (Greenwood-Van Meerveld and Johnson, 2017). Allostasis (from allostatic load to allostatic states) leads to decreased glutamate levels, an effect also observed in over-stressed rats (Ullmann et al., 2019). This research shows that allostasis may be a protective mechanism in rats for adapting to chronic stress (Ullmann et al., 2019). A recent study (Greenwood-Van Meerveld and Johnson, 2017) employed a rodent neuropathic pain model to assess the long-term impact of chronic pain on the hypothalamic pituitary adrenal (HPA) axis and limbic system. The results suggest that increased nociceptive sensitivity during chronic pain is associated with alterations in the limbic system, but is dissociated from HPA axis activation (Ulrich-Lai et al., 2006). This influence of long-term stress on nociception has been found to be relevant for numerous painful pathologies (Bardin et al., 2009). The effects of protracted or intermittent stress from daily, one hour restraint periods in cylinders, 4 days per week, over 5 weeks, on eight models of hyperalgesia and allodynia in rats was assessed. Their results showed that chronic stress can induce or trigger hyperalgesia and allodynia. As many conditions are characterized by hyperalgesia and allodynia, there is a need for a pre-clinical model integrating both chronic pain and stress. A model of prolonged or intermittent restraint stress is important to consider when investigating the mechanisms linking stress and chronic pain, and could provide insight to assessing the potential therapeutic efficacy of drugs targeted against painful pathologies with co-morbid stress (Bardin et al., 2009).

### Conclusion

Animal models confirm the complexity of the relationship between chronic pain and stress. Further research is needed to translate these animal findings to clinical populations across ages, sex, and pain conditions. Additionally, these animal models do not ultimately define how much stress is needed to contribute to pain presentation, nor do they define the specific impact of the timing, severity, or type of stress, and alternatively, if there is a healthy or protective dose of stress that could buffer or prevent the onset of chronic pain. Further animal research and pre-clinical models should explore resiliency and protective factors and how they may mediate the relationship between stress and chronic pain.

## Quantitative Sensory Testing, Acute Stress, and Chronic Pain

Quantitative sensory testing (QST) is used to assess responses to standardized noxious stimuli in a controlled laboratory setting. Research using QST has highlighted variability in pain sensitivity and pain modulation as a putative phenotypic contributor to the risk for development of chronic pain (Hansson et al., 2007).

### Acute Stress on Pain Perception and Sensory Functioning in Healthy Adults

The basis of individual differences in pain perception and neural responses to pain are not entirely understood. However, the neurophysiological mechanisms that regulate pain perception are influenced by the acute stress response. A review of 208 laboratory studies (Dickerson and Kemeny, 2004) assessing whether acute psychological stressors stimulate cortisol activation indicated that these acute psychological stressors do elicit cortisol activation, but certain stressors affect the HPA axis differently. Performance tasks with elements of social-evaluative threat and uncontrollability resulted in significant increases in cortisol levels and adrenocorticotropin hormone release (van den Bos et al., 2009; Engert et al., 2016; Goodman et al., 2017; Kluen et al., 2017).

It has been found that acute psychosocial stress has little effect on pain sensitivity, but significantly decreases an individual’s ability to regulate pain (Ahmad and Zakaria, 2015). The impact of acute psychosocial stress on heat pain perception was explored in a sample of healthy controls (Caceres and Burns, 1997) with results indicating that acute psychosocial stress did not impact heat pain threshold but slightly increased heat pain tolerance. Two studies have examined how pain perception and sensory functioning are influenced by acute stress in a healthy population. Geva et al. (2014) sought to examine the effect of acute stress on pain perception. The authors found that stress did not affect pain threshold and pain intolerance. However, stress did result in an increase in temporal summation of pain and a decrease in conditioned pain modulation, limiting the ability of participants to inhibit pain under acute psychological stress. Taken together, the results support the concept that the sensitivity to pain is not affected when an individual is exposed to acute psychosocial stress; however, the ability to modulate pain in a dose-response manner is significantly reduced. Considering the highly mixed results on the effect of acute stress on pain perception, it appears the type of stress and the magnitude of its appraisal determines its interaction with the pain system (Geva et al., 2014). Similarly, Gaab et al. (2016) sought to explore the impact of acute psychosocial stress on heat pain perception in a healthy sample. Results showed that acute psychosocial stress did not impact heat pain threshold but did slightly increase heat pain tolerance, suggesting that psychosocial stress is selectively analgesic for heat pain tolerance. To our knowledge, there is no published study investigating the effects of acute stress and pain perception and sensory functioning in children and adolescence, which would be an important area of inquiry.

### Acute Stress on Pain Perception and Sensory Functioning in Populations With Chronic Pain

Quantitative sensory testing has been used to measure sensory functioning and pain perception in several chronic pain populations. Groups of individuals with fibromyalgia (Crettaz et al., 2013; Coppieters et al., 2016; Wodehouse et al., 2018), chronic type headache (Cathcart et al., 2008; Cathcart et al., 2012; Defrin, 2014), whiplash disorders (Scott et al., 2005; Häggman-Henrikson et al., 2013), musculoskeletal pain (Paananen et al., 2015; Clark et al., 2017), menstrual pain (Slater et al., 2015; Payne et al., 2019), and irritable bowel syndrome (Murray et al., 2004) have previously undergone QST after exposure to acute psychological stress. A majority of these studies examined the relationship between acute stress and pain perception in individuals with chronic pain versus healthy controls. Specifically it has been demonstrated that patients with fibromyalgia (Crettaz et al., 2013; Coppieters et al., 2016) and chronic type headaches (Cathcart et al., 2008) respond differently to acute stress compared to healthy individuals. Specifically, (1) stress may have a negative impact on pain modulation in patients with chronic pain but not in healthy controls or patients with acute pain conditions (Coppieters et al., 2016); (2) acute stress can result in an increase in sensitivity to pressure pain only in chronic pain, and not in healthy controls (Crettaz et al., 2013); and (3) stress has a more significant hyperalgesic effect on cephalic pressure pain sensitivity in people with chronic pain than in healthy individuals (Cathcart et al., 2012). Additionally, differences were found between subsets of the chronic pain populations. Cognitive and somatic anxiety, fear, and avoidance were strongly correlated with pain tolerance in women with chronic type headache but not in women with migraines. This suggests that headache or pain frequency is one factor mediating the relationship between fear of pain and pain tolerance, which may help to explain why the relationship between pain perception and pain tolerance differs in people who experience more pain more often (Bishop et al., 2001).

### Conclusion

The question of why stress causes pain thresholds to decrease in certain people could be answered by examining changes in physiology. Alterations in physiological levels may modulate the impact of stress on pain. al’Absi and Petersen (2003) concluded that blood pressure levels mediated the effect of stress on pain ratings. They also noted that women with lower blood pressure had higher reports of pain, suggesting that men and women respond differently to acute stress due to different physiological changes. Similarly, Caceres and Burns (1997) found that men and women with high mean arterial pressure who were stressed before the cold pressor test had lower pain threshold and tolerance during the test than low mean arterial pressure reactors in the same conditions (Coppieters et al., 2016). Changes in physiological reactivity during stress exposure may determine how sensitive people are to pain and thus should be considered when investigating the impact of stress on the pathophysiology of chronic pain. Whereas acute stress often results in analgesia, chronic stress can trigger hyperalgesia/allodynia.

## Chronic Pain, Chronic Stress, and Allostatic Load

The association of between chronic stress and chronic pain has been historically assumed to be psychological, however, increasing recent research suggests physiological mechanisms may be relevant.

### Neurobiology of Chronic Stress

Research suggests that stress can mitigate the harmful effects of pain on the corticolimbic system (Vachon-Presseau, 2018). The resulting disturbance in equilibrium of these brain circuits have significant consequences both for chronic pain and for the normal regulation of the stress response. These effects are primarily through feedback mechanisms controlling the HPA axis. Much of the previous research has focused on the effects of stress on the regulation of the HPA axis, although inconsistencies in the direction of the effect (Fuentes and Christianson, 2018) (hypoalgesic versus hyperalgesic) have made the extent and manner of impact on chronic pain unclear. Chronic pain patients have been shown to exhibit faulty adaption stress responses, including both normal experiences and in response to pain (Vachon-Presseau, 2018). Whether the weakened response is damaging or adaptive remains unknown, but is important to consider because lower cortisol levels have been reported in individuals with chronic pain (Trickett et al., 2010). Research has also found that those who showed a greater cortisol response reported less pain unpleasantness and showed reduced activation in the nucleus accumbens, mid-cingulate cortex, and posterior insula during the painful stimulus (Vachon-Presseau et al., 2013). These brain regions are involved in cognitive modulation of pain and interact with the descending inhibitory pain pathway (Apkarian, 2010), thus mediating the stress-induced hypoalgesia (Butler and Finn, 2009). Chronic stress also appears to lead to a number of structural neurobiological changes related to pain processing, including reduced corpus callosum size, and decreased development of the left neocortex, hippocampus, and amygdala (Teicher et al., 2003). Psychological factors mediating altered pain processing have been shown in a range of psychological domains, including depression, anxiety, somatization, anger/hostility, self-efficacy, self-esteem, and general emotional functioning in people with chronic pain (Simons et al., 2014).

## What Are We Missing in Stress and Chronic Pain Models?

Although the relationship between chronic pain and stress is widely accepted, the interacting underlying biological mechanisms involved are less understood. A multidimensional model for considering the relationship of stress and chronic pain, along with the changing culture and mediating factors as environmental stress specifically among racial and ethnic minorities, and the impact of chronic pain remediations on stress.

## Role of Chronic Environmental Stress Among Racial and Ethnic Minorities

Stress is a consequence of racial and ethnic health disparities with the connection between stress and morbidity and mortality demonstrated across a variety of studies (Acabchuk et al., 1982; Berger and Sarnyai, 2015). Therefore, it is thought that greater exposure to stress over the life course increases susceptibility to morbidity and mortality among members of minority groups (Mocayar Marón et al., 2019). There is evidence of bias in pain management treatment, with White patients receiving higher levels of pharmacological pain treatment compared with Black patients (Drwecki et al., 2011). The presence of stress results in molecular, cellular, and neural-circuit level changes. Stress triggers activation of molecular processes that tag genes with “epigenetic marks” that result in long-lasting changes in how the molecular machinery of those neurons is expressed. Epigenetic marks can last for months, years, or perhaps even lifetimes, and the gene expression that results from these marks change how neurons respond to their environment as well as future adversity or stress (Kanherkar et al., 2014). The role of environmental stress among racial and ethnic minorities and the impact on the exacerbation of pain needs to be investigated.

### Psychopharmacology, Stress, and Chronic Pain

While opioids and NSAIDS are used to treat acute pain (Schug and Goddard, 2014) and anti-convulsants and/or tricyclic anti-depressants (Finnerup et al., 2015) are used for treating neuropathic pain, pharmacological interventions for the treatment of co-morbid stress and pain are elusive. Sieberg et al. (2018) offered mechanistic evidence as to why many anxiolytic drugs may also effective with neuropathic pain medications. Such drugs can increase norepinephrine in the spinal cord, which alleviates pain (Pitzer et al., 2016); however, concluding from the results, it is possible that the reduction of high anxiety-like levels alone also contributes to the efficacy of these drugs. Anxiolytics coupled with neuropathic pain medication may reduce comorbid anxiety-like behavior as well as decreased pain-like behavior. Although stress and anxiety differ, researchers should consider these findings when moving forward with stress and pain research. Many patients suffering from comorbid chronic pain and stress would benefit from additional research investigating the impact of existing neuropathic and anxiolytic medications. Additionally, further elucidating the relationship between pain and stress can assist in the development of more targeted therapies. Established and valid biomarkers for pain and stress can help to determine and predict if a patient will respond better to specific treatments, thus improving the quality of life and decreasing pain of those who suffer from co-occurring chronic pain and stress.

### Conclusion

There are many mediating factors contributing to the complex relationship of chronic pain and chronic stress. The role of environmental stress among racial and ethnic minorities and the impact on the exacerbation of pain needs to be further investigated. While numerous biomarkers including genetic (Cronin et al., 2018), molecular (König et al., 2017), neural (Smith et al., 2017), inflammatory (Arif-Rahu et al., 2012), and biobehavioral (Arif-Rahu et al., 2012) have been identified as contributing to chronic pain, there are currently no valid or reliable biomarkers for chronic pain. One biomarker alone is unlikely to fully explain the complexity of pain syndromes that are influenced by many factors, such as different types and levels of sensitivity to chronic stress. A multisystem approach is needed to improve the diagnosis, prognosis, and the evaluation of treatment responses, and inform drug development, as well as to elucidate the relationship between stress and chronic pain.

## Conclusion

Stress has multifaceted effects on chronic pain. Chronic stress, impacting an individual’s capacity to cope, affects the brain. Similarly, chronic pain is widely considered a disease of the CNS. Stress can be a powerful inhibitor of nociception and inflammation but also contributes to enhanced pathological states including the initiation and maintenance of chronic pain (Vachon-Presseau, 2018). Understanding the complex relationship between stress and chronic pain requires interdisciplinary collaboration, translational models, and consideration of biopsychosocial factors, which will hopefully result in improved, standardized care.

**FIGURE 1:**
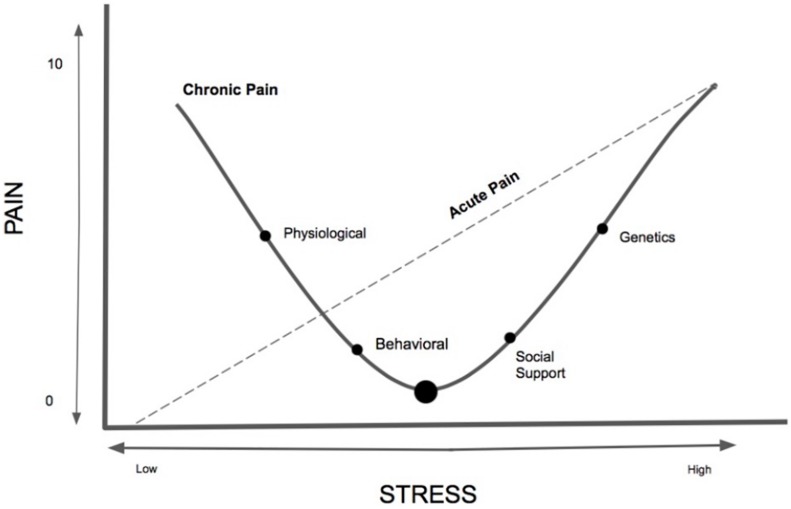
The newly conceptualized Pain-Stress Model, adapted from the Yerkes–Dodson Law, is a proposed curvilinear and linear model describing the multifaceted relationship between chronic pain and stress. The *y*-axis represents the Numerical/numeric Rating Scale (NRS), a standardized pain intensity assessment (mild: 1–3; moderate: 4–6; severe: 7–10 (Rivat et al., 2010). The Pain-Stress Model provides a framework for addressing the multi-factorial nature of stress and chronic pain. Approaches to studying multiple, interacting physiological systems and molecular pathways is needed for the development of translatable biomarkers that would facilitate the study of stress responses, resilience, and vulnerability across both human and animal studies. Not all individuals are susceptible to environmental stress factors, however, this model portrays the curvilinear relationship between chronic pain and stress and the linear relationship between stress and acute pain – it is unknown what amount of acute or chronic stress is protective for the nervous system of chronic pain patients and when it transitions to a damaging effect.

It is unclear the amount, timing, severity, and type of stress needed to contribute, maintain and exacerbate chronic pain and whether alternatively there is a healthy or protective dose of stress that could buffer or prevent the onset of chronic pain. Based on the existing scant literature on stress and chronic pain, this seemingly paradoxical relationship can be better understood using our proposed theoretical model, labeled the Pain-Stress Model, adapted from both the Yerkes–Dodson Law (Diamond et al., 2007) and the biological reactivity to psychological stressors model (Boyce and Ellis, 2005; Figure 1). We theorize that chronic pain has a curvilinear relationship with chronic stress and a relatively linear relationship with acute stress, with various biopsychosocial mediators and moderators serving as both risks and protective factors. The Pain-Stress Model suggests that some stress is protective against the development of chronic pain; however, at a point the amount of and severity of the stress will become damaging. Moreover, we suggest that this relationship has a bidirectional effect, with chronic pain also negatively impacting stress levels. We propose that this model be empirically studied in order to further untangle the complicated relationship between stress and pain.

## Author Contributions

CL conducted the literature review and wrote the majority of the manuscript with mentoring, editing, and guidance from CS. CS conceptualized the Pain-Stress Model with input from CL.

## Conflict of Interest

The authors declare that the research was conducted in the absence of any commercial or financial relationships that could be construed as a potential conflict of interest.
